# ACKNOWLEDGEMENT TO AD HOC REVIEWERS: 2019

**DOI:** 10.5327/Z167944352019LIST

**Published:** 2019-12-01

The editors of the Brazilian Journal of Occupational Medicine (RBMT), of the National Association of OccupationalMedicine (ANAMT) are grateful for the qualified and voluntary work offered by the professional experts who servedthroughout the year 2019 asad hocreviewers, which, with integrity, independence and competence, made it possibleto maintain and improve the double blind peer review system, one of the fundamental pillars of the good scientificjournals. Thank you very much!

Adilson Morato Filho

Adriana Jardim Arias Pereira

Adriane Mesquita de Medeiros

Adriano Drummond

Alessandra Mussi Ribeiro

Alexandre Crespo Pinto

Alfredo Almeida Pina-Oliveira

Alice de Oliveira de Alchorne

Aline Silva-Costa

Álvaro Francisco Lopes de Sousa

Ana Carolina Guidorizzi Zanetti

Ana Claudia Souza Vazquez

Ana Isabel Dias da Silva

Ana Luiza Castro Fernandes Villarinho

Ana Paula Scalia Carneiro

Andréa Aparecida Luz

Andrea Maria Silveira

Angela Helena Marin

Angelle Aragonez Essado Jacomo

Antonia Regina Ferreira Furegato

Arthur Brito Marcelino

Barbara Cecconi Deon

Bianca Anne Mendes de Brito

Bruna Xavier Morais

Caio Bezerra Souto Maior

Camila Castro

Camila Rego Amorim

Carlos António Laranjeira

Carlos Botazzo

Carlos Henrique de Freitas Lima

Charli Sargent

Claudete Moreschi

Claudia Esteban

Claudia Maria Bogus

Cleonice Andréa Alves Cavalcante

Cleucimara Aparecida Camilo

Cristiane Gallasch

Cristiane Rapparini

Cristiano Barreto de Miranda

Cristine Scattolin Andersen

Dafne Braga Diamante Leiderman

Daniele Maciel Pimentel

Danielle Cristina Guimarães da Silva

Danniela Britto de Carvalho

Deciane Pintanela de Carvalho

Diogo Sousa Lemos

Dvora Joveleviths

Eduardo Algranti

Eduardo Branco de Sousa

Eduardo de Paula Lima

Eduardo Garcia

Eduardo Myung

Elamara Marama Vieira

Eliane Florêncio Gama

Elias Barbosa de Oliveira

Ema Sacadura Leite

Emil Kupek

Ester Paiva Souto

Evelin Daiane Gabriel Pinhatti

Fabio José da Silva

Fabrício Augusto Menegon

Fernanda Monteiro Dias

Fernanda Moura D’Almeida Miranda

Fernanda Netto

Fernanda Oliveira Ulguim

Fernando Akio Mariya

Fernando Ribas Feijó

Flávia Souza e Silva de Almeida

Flavio Liberali Magajewski

Flávio Tocci

Florentino Serranheira

Gilson José Allain Teixeira Junior

Giselle Santana Dosea

Glenda Blaser Petarli

Henrique César Santejo Silveira

Henrique Luiz Monteiro

Iara Sayuri Shimizu

Iracema Viterbo Silva

Isabela Ballalai

Isabela Dias Lauar

Isabelle Pereira Soares Mariz

Jacqueline Flores de Oliveira

Jaqueline Brito Vidal Batista

Jefferson Benedito Pires de Freitas

Jefferson Peixoto da Silva

João Carlos Lozovey

José Francia

José Geraldo Mill

Júlia Trevisan Martins

Julianne Cristine Ferreira

Julizar Dantas

Karina Araujo Pinto

Karol Fireman Farias

Kátia Sheylla Purim

Leni de Lima Santana

Lúcia Rosa de Carvalho

Luciana Ferreira Campos Vasconcelos

Luciana Zaranza Monteiro

Luciane Albuquerque Sá de Souza

Luciane Peter Grillo

Luciano Garcia Lourenção

Luiz Antonio Nogueira Martins

Luiz Felipe Rigonatti

Luiz Guilherme Grossi Porto

Luiz Menna Barreto

Luiz Sérgio Silva

Manoela Lopes

Mara Alice Conti Takahashi

Mara Gandara

Marcelo Monteiro Ferreira

Marcelo Moreira Corgozinho

Márcia Astrês Fernandes

Márcia Valéria Rosa Lima

Marcos André de Matos

Marcos Brioschi

Marcos Campello Baptista

Marden Samir Santa Marinha

Maria Amélia Ximenes

Maria do Carmo Fernandez Haddad Lourenço

Maria Juliana Moura Correa

Maria Lúcia do Carmo Cruz Robazzi

Marina Favrim Gasparini

Marina Pereira Rezende

Marluce Rodrigues Godinho

Melissa Andreia de Moraes Silva

Michele Cristiene Nachtigall Barboza Martinato

Michele Nusbaum

Milena Cordeiro

Milton Helfenstein Júnior

Mirian Werba Saldanha

Nádia Bruna da Silva Negrinho

Nágila Soares Xavier Oenning

Naiá Ortelan

Paloma de Souza Cavalcante Pissinati

Patrícia Dalagasperina

Paulo Antonio Barros Oliveira

Paulo de Oliveira Perna

Paulo Roberto Veiga Quemelo

Rafael Augusto Tamasauskas Torres

Rafael Haeffner

Rafaela Andolhe

Ramires Alsamir Tibana

Raphael Mendonça Guimarães

Renata Cavalcanti Carnevale

Rita Zurbriggen

Robson da Fonseca Neves

Robson Spinelli

Rodrigo Bornhausen Demarch

Rogério Muniz de Andrade

Rosa Amélia Andrade Dantas

Rosana Lazzarini

Rosane Harter Griep

Rubens Bedrikow

Sâmea Cristina Santos Gomes

Sandra Maria Gasparini

Selma Lancman

Sergio Roberto de Lucca

Sergio Valverde Marques dos Santos

Shamyr Sulyvan Castro

Silmar Maria Silva

Silvia Helena Henriques

Silviamar Camponogara

Suleima Pedroza Vasconcelos

Taianah Almeida Barroso

Tânia Adas Saliba

Tania Magnago

Tatiana Frederico de Almeida

Tereza Raquel Ribeiro de Sena

Thais Martins da Silva

Ubirani Barros Otero

Ubiratan de Paula Santos

Valéria Masson

Vera Regina Lorenz

Victor Wünsch Filho

Vilma Leyton

Viviana Gómez Sánchez

Volney de Magalhães Câmara

Wellington Segheto

